# Important factors associated with sick leave after allogeneic haematopoietic stem cell transplantation—a 1-year prospective study

**DOI:** 10.1007/s11764-020-00986-5

**Published:** 2021-01-08

**Authors:** Linda Eriksson, Agneta Wennman-Larsen, Karin Bergkvist, Per Ljungman, Jeanette Winterling

**Affiliations:** 1 Department of Neurobiology, Care Sciences and Society, Division of Nursing, Karolinska Institutet, Alfred Nobels allé 23, C4, 141 52 Stockholm, Sweden; 2 Department of Haematology, Karolinska University Hospital, Stockholm, Sweden; 3 Department of Clinical Neuroscience, Division of Insurance Medicine, Karolinska Institutet, Stockholm, Sweden; 4 Sophiahemmet University, Stockholm, Sweden; 5 Department of Medicine Huddinge, Division of Haematology, Karolinska Institutet, Stockholm, Sweden; 6 Department of Cellular Therapy and Allogeneic Stem Cell Transplantation, Karolinska University Hospital Huddinge, Stockholm, Sweden

**Keywords:** Allogeneic haematopoietic stem cell transplantation, Sick leave, Sickness absence, Return to work

## Abstract

**Purpose:**

This study examines sick leave (SL) and factors associated with full-time SL 1 year after allogeneic haematopoietic stem cell transplantation (allo-HSCT) in patients of working age from 2009 to 2016 (*n* = 122).

**Methods:**

Questionnaire data were collected on admission to the allo-HSCT unit, at 7 months and 1 year after allo-HSCT. Associations between factors and SL were analysed using logistic regression analyses.

**Results:**

One year after allo-HSCT, 76% of participants were on SL, with 36% on full-time SL. In univariable analyses, chronic graft-versus-host-disease (cGvHD) (OR 3.07; 95% CI 1.34–7.07; *p* = 0.01), having symptoms of depression at 7 months (OR 4.81; 95% CI 1.69–13.69; *p* = 0.00) and low levels of vocational satisfaction at 7 months after treatment (OR 3.27; 95% CI 1.27–8.41; *p* = 0.01) were associated with full-time SL 1 year after allo-HSCT. cGvHD (OR 3.43; 95% CI 1.35–8.73; *p* = 0.01) and having symptoms of depression at 7 months after allo-HSCT (OR 3.37; 95% CI 1.2–11.58; *p* = 0.02) remained significant in multivariable analysis.

**Conclusion:**

The majority of allo-HSCT survivors were on SL 1 year after treatment, and cGvHD, low vocational satisfaction and depressive symptoms were associated with full-time SL 1 year after allo-HSCT.

**Implications for Cancer Survivors:**

Healthcare professionals need to be observant of and manage the consequences of cGvHD and patients’ symptoms of depression in order to support them appropriately in their return-to-work process.

## Introduction

An allogeneic haematopoietic stem cell transplantation (allo-HSCT) is a procedure where healthy haematopoietic stem cells from a donor are infused to a recipient to replace stem cells that are defect from the underlying disease or treatment in the hope that the recipient will recover the ability to produce their own healthy haematopoietic stem cells. Prior to allo-HSCT, patients receive chemotherapy and/or radiation therapy in order to suppress their underlying malignancy and facilitate the engraftment of the new stem cells [1]. It is a demanding and intensive treatment that often results in many long-lasting disease and treatment-related difficulties that negatively affect patients health [2, 3], making those who are of working age unable to work or study [4]. After an allo-HSCT, an important goal is to return to normal life, which includes return to work (RTW) as it can represent a return to normality [5] and often increases quality of life [6].

Research has shown that factors associated with a lower likelihood of RTW for allo-HSCT survivors include female gender, older age, poorer health and worse physical functioning [4, 7] along with fatigue, pain and lower quality of life [6, 8]. The most common late complication of allo-HSCT is chronic graft-versus-host-disease (cGvHD), which affects 20–50% of survivors [9]. GvHD is an immune reaction mediated by T cells between the donated stem cells and the recipient [1]. The first phase of this immune response, acute GvHD (aGvHD), causes direct tissue damage in varying degrees and generally manifests in a set of organs (i.e. skin, liver and gastrointestinal tract) [1]. The second phase, cGvHD, tends to be more delayed in its presentation and involves a broader set of organs that can bear strong resemblances to autoimmune disorders [1]. Whether cGvHD has an effect on RTW is not clear as some studies state there is no association [7, 10] whereas other studies suggest that there is a relationship between the two [9, 11]. These mixed results can depend on differences in study design, sample size and treatment regimes. Due to the psychological challenges associated with allo-HSCT, patients are at risk of anxiety and depression [12–15], and previous research has found that patients with a haematological malignancy who showed symptoms of anxiety were less likely to RTW [8]. Satisfaction with one’s vocational and financial situation may also influence RTW. Earlier studies on non-haematological patients indicate that low satisfaction with one’s vocational [16–18] and financial situation [19] is associated with reduced RTW.

Long periods of sick leave (SL) can have a negative impact on areas such as health, work, social relationships and financial situation causing changes in self-image, feelings of exclusion and social stigma [20–25]. The prerequisites of being on SL differ between countries. In Sweden, everyone with an income from work, unemployment benefits, studies or parental leave benefit is covered by the national social insurance and qualifies for sickness benefits if unable to work due to disease or injury that reduces their work ability by at least 25%. The levels of compensation can be 25, 50, 75 or 100% of regular work hours depending on the reduction in work ability, covering approximately 80% of lost income up to a certain level. Identifying allo-HSCT patients at risk of long-term SL may help improve rehabilitation and increase quality of life*.* Hence, the aim of this study was to examine SL 1 year after allo-HSCT and its association with demographic, medical, and psychological factors, together with vocational and financial satisfaction.

## Materials and methods

This is a longitudinal, prospective single-centre study using data collected at the time of allo-HSCT, 7 months and 1 year after allo-HSCT.

### Sample

**Fig. 1:**
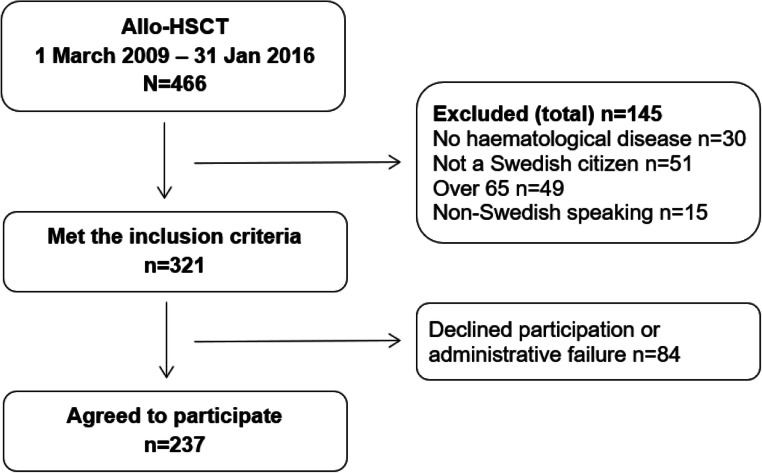
Enrolment of study participants

All consecutive adult patients admitted for allo-HSCT between March 2009 and January 2016 were screened, and those fulfilling the inclusion criteria were approached. Inclusion criteria were haematological disease, age 18–65 years, able to understand Swedish and living in Sweden (Fig. 1). In total, 321 patients met the inclusion criteria of whom 237 (74%) agreed to participate, while the remainder (*n* = 84) either declined participation or were not invited to participate. The reasons for the latter were not recorded but are likely to have been due to the staff being too busy or the patient too sick. At baseline, 237 questionnaires were handed out and 194 responded (82% response rate). Only those who responded to the questionnaire at all three time points (*n* = 136) and did not report early retirement or disability pension (*n* = 14) at any time point were included (*n* = 122).

### Procedure

When admitted to the allo-HSCT unit, eligible participants were given both verbal and written information, including a consent form. Baseline questionnaire data were collected on admission to the allo-HSCT unit, and follow-ups were at 7 months post allo-HSCT and close to the 1-year medical follow-up visit. Follow-up questionnaires were sent by post. If it was not returned, the participants were reminded twice by phone. Questionnaires were sent out at all time points to all participants, regardless of whether they had answered the previous questionnaires unless the participant had declined or died.

### Data collection

Medical data was retrieved from the participants’ medical records. Data regarding demographics, psychological factors, life satisfaction and sick leave were gathered from a questionnaire originally developed for breast cancer patients [26], which has previously been used on allo-HSCT patients [4, 27].

Sick leave was reported by the question “Are you on sick leave now?” with the response alternatives; “no” and “yes”. Participants also responded to questions about “Full-time sick leave” or “Part time sick leave” with space to write the percentage of SL. Equivalent data were gathered from the medical records of participants who did not reply to this question. In this study, participants were considered as being on SL if they had been sick listed for at least 90 continuous days and full-time SL if they reported being on SL > 75% of a full-time position.

Anxiety and depression were measured using the validated self-assessment Hospital Anxiety and Depression Scale (HADS) [28]. The scale consists of two subscales (anxiety and depression) with seven items each. Each item is scored from 0 to 3. Total scores for each subscale are calculated (ranging from 0 to 21). A higher score indicates higher levels of anxiety and/or depression. The cutoff value for symptoms of either anxiety and/or depression was ≥ 8 [29].

Life satisfaction was measured using the validated Life Satisfaction Checklist-11 (LiSat-11) [30]. Two items were used; satisfaction with vocational situation and satisfaction with financial situation. The participants rated each item on a 6-point scale from “very dissatisfied” to “very satisfied”. The answers were dichotomised into “low satisfaction” (“very dissatisfied to “rather satisfied”’) and “high satisfaction” (“satisfied” to “very satisfied”).

### Statistical analyses

Potential statistical differences between baseline respondents and non-respondents were analysed using either independent *t* tests or two-sided *χ*2-tests depending on the data level. Missing data in instruments based on sum scores, i.e. HADS, were replaced using person-mean imputation [31] if missing data did not exceed 20% for each scale [32]. Odds ratios (OR) with 95% confidence intervals (CI) were calculated using univariable logistic regression analyses between each factor and full-time SL 1 year after allo-HSCT. The significance level was set to *p* < 0.05. Factors analysed were demographic (gender, age, living situation, education), medical (diagnosis, conditioning, total body irradiation, relapse, presence of cGvHD), psychological (symptoms of anxiety and depression) and satisfaction with vocational and financial situation. A multivariable logistic regression was then performed where all factors with a *p* value ≤ 0.05 in the univariable analyses were included. The statistical calculations were conducted using SPSS 24.0 (IBM, Chicago, IL, USA).

## Results

There were no statistically significant differences between baseline respondents (*n* = 194) and non-respondents (*n* = 43) regarding age and diagnosis, but a statistically significant difference was found regarding gender with females participating to a higher degree (*X*^2^(1) = 4.6, *p* = 0.03).

Medical and sociodemographic data are presented in Table 1. The proportion having symptoms of anxiety did not differ much between baseline and 7 months post-allo-HSCT, with 25% of the participants scoring above the cutoff value at baseline and 24% at 7 months. The proportion having symptoms of depression decreased slightly from baseline (21%) to 7 months post-treatment (17%). At baseline, approximately half of the participants reported low levels of satisfaction (i.e. very dissatisfied to rather satisfied) with their vocational (55%) and financial situation (49%). At 7 months after allo-HSCT, the number of participants reporting low levels of satisfaction increased for both vocational (71%) and financial situation (63%).

**Table 1:** Baseline demographic and medical data of the participants in the study (*n* = 122)

Demographic data		*n* (*%*)
Gender	Women Men	49 (40) 73 (60)
Age	Younger (< 52 years)^1^ Older (≥ 52 years)	58 (48) 64 (52)
Marital status	Married Not married	77 (63) 40 (33)
Living situation	Living alone Living with someone	17 (13) 102 (87)
Children	Yes No	93 (76) 26 (21)
Education	Lower (elementary/secondary school) Higher (college/university)	45 (38) 74 (62)
Country of birth	Sweden Abroad	107 (88) 11 (9)
Medical data		*n* (*%*)
Diagnosis	Acute leukaemia Chronic leukaemia Lymphoma Plasma cell disorders Myelodysplastic syndrome Myeloproliferative neoplasia Other^2^	51 (42) 24 (20) 10 (8) 10 (8) 16 (13) 7 (6) 4 (3)
Conditioning	Myeloablative Reduced intensity	84 (69) 38 (31)
Total body irradiation	Yes No	86 (70) 36 (30)
Clinical status at allo-HSCT	Complete remission Partial remission Chronic phase No response Stable disease Untreated Not applicable	74 (61) 24 (20) 9 (7) 2 (2) 6 (5) 3 (2) 4 (3)
Stem cell source	Bone marrow Peripheral blood	14 (12) 108 (88)
Type of donor	Identical sibling, syngeneic Unrelated^1^ Mismatched relative	41 (34) 78 (64) 3 (2)
Retransplantation (during 1st year after allo-HSCT)	No Yes	116 (95) 6 (5)
cGvHD (at 1 year)	No Mild Moderate Severe	90 (74) 20 (16) 12 (10) -
Relapse (during 1st year after allo-HSCT)	No Yes	108 (88) 14 (12)
^1^Divided by the median. ^2^Immunodeficiency, SAA, ^2^matched unrelated, mismatched unrelated, unrelated		

### Description of SL from diagnosis to 1 year after allo-HSCT

**Fig. 2:**
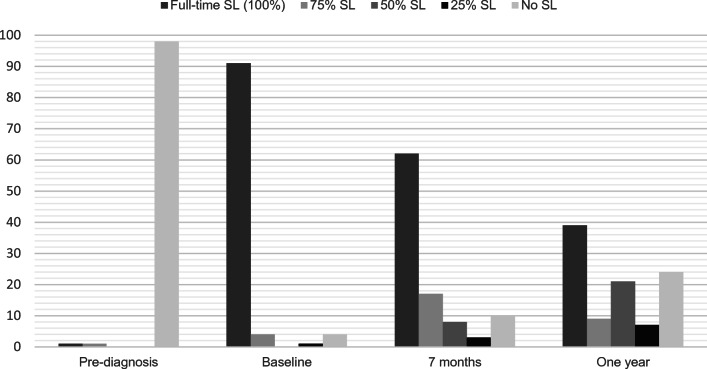
Proportions of participants (%) on SL at the different time points (*n* = 122)

Before diagnosis of their haematological disease, 98% of participants were working or studying full-time and only 2% reported being on SL. At baseline, when they were admitted for the allo-HSCT, 94% were on SL. Those not on SL at baseline stated that they were self-employed. Seven months after allo-HSCT, 90% of the participants were on either full- or part-time SL, which dropped to 76% 1 year after allo-HSCT. One year after treatment, 39% of all participants were on full-time SL. The proportions of levels of compensation and those on full-time SL (> 75–100%), part-time SL (1–75%) or no SL from pre-diagnosis to 1 year after allo-HSCT can be seen in Fig. 2.

### Factors associated with full-time SL 1 year after allo-HSCT

In univariable analyses, the factors associated with full-time SL 1 year after allo-HSCT were cGvHD (OR 3.07; 95% CI 1.34–7.07; *p* = 0.01), having symptoms of depression at 7 months (OR 4.81; 95% CI 1.69–13.69; *p* = 0.00) and low levels of satisfaction regarding vocational situation at 7 months (OR 3.27; 95% CI 1.27–8.41; *p* = 0.01) (Table 2). Gender, age, education, living situation, diagnosis, conditioning treatment, total body irradiation, relapse, having symptoms of anxiety and satisfaction with financial situation were not associated with full-time SL 1 year after allo-HSCT. The only factors independently associated with full-time SL 1 year after allo-HSCT in the multivariable analysis were cGvHD (OR 3.43; 95% CI 1.35–8.73; *p* = 0.01) and having symptoms of depression at 7 months (OR 3.37; 95% CI 1.2–11.58; *p* = 0.02) (Table 2).

**Table 2:** Descriptions of proportions of demographic, medical and psychological factors along with satisfaction with vocational and financial situation and odds ratio (OR) with 95% confidence interval (CI) for those on full-time SL (*n* = 48) at the 1-year follow-up among all participants (*n* = 122)

Independent variable		Total (*n* = 122) *n* (%)	Full-time SL (*n*=48) *n* (%)	Univariable analysis OR (95% CI)	*p* value	Multivariable analysis OR (95% CI)	*p* value
Demographic factors							
Gender	Female Male (ref)	49 (40) 73 (60)	20 (41) 28 (38)	1.11 (0.53–2.32)	0.79		
Age	Older Younger (ref)	64 (52) 58 (48)	26 (41) 22 (38)	1.12 (0.54–2.32)	0.76		
Education	Lower Higher (ref)	45 (38) 74 (62)	19 (42) 28 (38)	1.2 (0.56–2.56)	0.64		
Living situation	Living alone Living with someone (ref)	17 (14) 102 (86)	8 (47) 39 (38)	1.44 (0.51–4.03)	0.49		
Medical factors							
Diagnosis	Acute leukaemia All others (ref)	53 (43) 69 (57)	18 (34) 30 (44)	0.7 (0.32–1.4)	0.29		
Conditioning	Myeloablative (MAC) Reduced intensity (RIC) (ref)	38 (31) 84 (69)	15 (40) 33 (39)	1.0 (0.46–2.21)	0.98		
Total body irradiation	Yes No (ref)	36 (30) 86 (70)	14 (39) 34 (40)	0.97 (0.44–2.16)	0.95		
cGvHD (at 1 year)	Yes No (ref)	32 (26) 90 (74)	19 (59) 29 (32)	3.07 (1.34–7.07)	0.01	3.43 (1.35–8.73)	0.01
Relapse (during 1st year after allo-HSCT)	Yes No (ref)	14 (11) 108 (89)	9 (64) 39 (36)	3.19 (1.00–10.18)	0.05		
Psychological factors							
Symptoms of anxiety at baseline	Case Non-case (ref)	29 (25) 87 (75)	15 (52) 31 (36)	1.94 (0.83–4.53)	0.13		
Symptoms of anxiety at 7 months	Case Non-case (ref)	28 (24) 90 (76)	12 (43) 34 (38)	1.24 (0.52–2.92)	0.63		
Symptoms of depression at baseline	Case Non-case (ref)	24 (21) 90 (79)	12 (50) 33 (37)	1.73 (0.7–4.28)	0.24		
Symptoms of depression at 7 months	Case Non-case (ref)	20 (17) 98 (83)	14 (70) 32 (33)	4.81 (1.69–13.69)		3.73 (1.2–11.58)	0.02
Vocational and economical satisfaction							
Vocational situation at baseline	Low High (ref)	63 (55) 52 (45)	28 (44) 18 (35)	1.51 (0.71–3.22)	0.29		
Vocational situation at 7 months	Low High (ref)	79 (71) 33 (29)	37 (47) 7 (21)	3.27 (1.27–8.41)	0.01	2.79 (1.0–7.75)	NS (0.05)
Financial situation at baseline	Low High (ref)	56 (49) 58 (51)	25 (45) 20 (35)	1.53 (0.72–3.26)	0.27		
Financial situation at 7 months	Low High (ref)	73 (63) 43 (37)	30 (41) 15 (35)	1.3 (0.6–2.85)	0.51		

*OR*, odds ratio; *CI*, confidence interval

## Discussion

This is the first longitudinal study focusing on SL during the first year after allo-HSCT. The results show that 39% of the participants were on full-time SL 1 year after allo-HSCT and that factors of importance for SL were symptoms of depression at the 7-month follow-up together with cGvHD 1 year after treatment.

Examination of SL during the first year after allo-HSCT revealed that almost all participants were on full-time SL at the time of allo-HSCT and although the percentage decreased over time, 39% were still on full-time SL 1 year after treatment. No studies investigating SL after allo-HSCT were found with which to compare the present results. However, a longitudinal study found that of patients who had undergone an autologous HSCT due to relapse after treatment for lymphoma, 30% remained on SL after the first year (part- or full-time SL unknown) (*n* = 164) [33]. Several studies have examined RTW among HSCT patients and estimates range from 20% to approximately 45% of patients returning to full-time work 1 year after transplantation in research including both autologous and allogeneic participants [10, 11, 7, 33]. Moreover, fewer allogeneic patients RTW within the first year after transplantation compared to autologous patients [11].

Comparing allo-HSCT patients with other cancer diagnoses can be problematic, as treatment regimens vary considerably, but studies have revealed that chemotherapy can significantly prolong SL and delay RTW [34–37]. However, in the present study, 76% were on either part- or full-time SL, which is a large proportion compared to, e.g. breast cancer patients in Sweden, where close to 30% were on SL (with less than 10% on full-time SL) 1 year after treatment [38]. Furthermore, being on SL for longer periods of time can in itself be associated with difficulties related to RTW among patients with different types of cancer diagnoses [39].

An explanation for the high proportions of being on SL in this study could be that clinicians recommend all allo-HSCT patients to take full-time SL for at least 6 months unless they can easily adapt their employment to reduce the risk of becoming infected with, for example, community acquired respiratory viruses such as influenza and respiratory syncytial virus. This recommendation is prolonged if at 6 months post-treatment a patient is still on full immunosuppression for GvHD. This is a unique situation not applicable to most other cancer patients, and the current Covid-19 pandemic has only increased the need for this type of self-protection from respiratory viruses in allo-HSCT patients [40].

None of the demographic factors investigated in this study were associated with full-time SL 1 year after allo-HSCT. However, sociodemographic factors have previously been shown to be associated with SL and RTW. In a large study including different types of cancer diagnoses, higher age and lower education predicted longer SL [41], while studies focusing on RTW among allo-HSCT survivors found that those of female gender and older age were less likely to RTW [7, 4], whereas no associations with educational level were found [7]. These differences may depend on when the measurements of RTW were done or be due to other factors being more important, as allo-HSCT survivors often face complications and a slow immune reconstitution [2, 3].

The medical factor cGvHD was significantly associated with full-time SL 1 year after allo-HSCT both in univariable and multivariable analyses, which is not surprising and in agreement with two earlier studies focusing on RTW [9, 11]. Previous studies show that survivors with cGvHD experience significant functional limitations in areas such as mental health [9, 12], quality of life [9, 42] and overall survival [43, 44]. Interestingly, relapse during the first year after allo-HSCT was not associated with full-time SL 1 year after transplantation. Previous research in autologous HSCT patients has found that relapse of the primary disease was associated with a lower likelihood of RTW in the first year after treatment [11] along with a decreased rate of RTW for patients who were on SL at the time of relapse [33]. The results suggest that those included in this study who relapsed during the first year after allo-HSCT were treated successfully, as they were able to participate in the study at the 1-year follow-up. Unsuccessful treatment of relapse after allo-HSCT often results in death, which in this study resulted in a lack of data at the 1-year follow-up on those most affected by relapse.

The present results show that having symptoms of depression at 7 months after treatment, but not at baseline, was associated with full-time SL 1 year after allo-HSCT. No previous studies have been found that specifically explore associations between depression and SL after allo-HSCT. However, although it is known that depression negatively affects RTW after injury or illness in general [45], this has not been shown among patients with a haematological malignancy [8]. Nevertheless, one study indicated that being unemployed is linked to depression after allo-HSCT [13]. It can be questioned whether having anxiety and depression symptoms make a person remain on SL or if SL itself increases the risk of developing these symptoms. Regardless of the cause, it is important to be aware that after allo-HSCT patients are at risk of anxiety, depression [14, 12, 15] and cGvHD [9] and that these appear to be interrelated as studies show associations between cGvHD and mental health [9, 12]. Thus, it is important to identify those who are at risk of depression. More in-depth, qualitative research would be of interest to further explore the ways in which depression can affect allo-HSCT survivors.

A factor associated with SL in the univariable analysis was low vocational satisfaction at 7 months, but not at baseline, after allo-HSCT. This is in line with studies of breast cancer patients, where low vocational satisfaction 6 weeks post-surgery predicted non-RTW 10 months after surgery [18]. Also, significant associations have been found between vocational dissatisfaction and long-term SL among non-cancer patients [16, 17]. Vocational satisfaction can be dependent on whether the patient has employment to return to as it is difficult to judge from the present results whether low satisfaction is due to SL or vice versa. An American study found that it is common for allo-HSCT patients to experience serious adverse financial consequences [46]. Therefore, it is worth noting that satisfaction with the current financial situation was not associated with full-time SL in the present study. This may be due to the Swedish context where the national social insurance system financially compensates patients for loss of earnings following injury or illness.

A strength of this study is the inclusion of longitudinal data and a relatively large sample size, considering that approximately only 400 allo-HSCT are performed annually in Sweden. However, it is still possible that a larger sample size might have yielded different results. A limitation is the lack of information regarding SL of those who were not invited to participate, especially as the reasons for not being included are unknown. Another strength is the use of clinically diagnosed cGvHD data, as many other studies only use self-reported data, which could be considered uncertain due to subjectivity. The lack of participants diagnosed with severe cGvHD means that those most affected by cGvHD were not included, possibly because they either dropped out or died. In the logistic regression analyses, the presence rather than the severity of cGvHD was analysed, which could have influenced the cGvHD results. However, analyses of the severity of cGvHD were also performed and showed similar results. In addition, the likelihood of participants being classified as having symptoms of depression might be overestimated in this study since symptoms of depression were measured using the HADS self-assessment scale and not diagnosed by a clinician. However, HADS has been found to have excellent case finding abilities in many settings [29]. In this study, data on SL is from the 1-year follow-up. A suggestion for further studies in this patient group would be to examine self-reported reasons for SL, and report on SL further along the trajectory than 1 year as earlier research has shown that RTW is a long process for this patient group [47].

In conclusion, this study appears to be the only study examining SL and factors associated with full-time SL 1 year after allo-HSCT. The results show that a large proportion of allo-HSCT survivors are still on full-time SL 1 year after treatment and that cGvHD and depressive symptoms may be associated with SL. These results suggest that it is important for health care staff to closely monitor and manage the consequences of cGvHD and be observant of and find appropriate care strategies for patients’ symptoms of depression in the aftermath of allo-HSCT to increase the likelihood of a successful RTW.

## Data Availability

The datasets generated and/or analysed during the current study are available from the corresponding author on reasonable request.
